# Acute pancreatitis induced by mycophenolate mofetil in a kidney transplant patient

**Published:** 2015-02-18

**Authors:** Behzad Einollahi, Fardin Dolatimehr

**Affiliations:** ^1^Nephrology and Urology Research Center, Baqiyatallah University of Medical Sciences, Tehran, Iran; ^2^Students’ Research Center, Baqiyatallah University of Medical Sciences, Tehran, Iran

**Keywords:** Kidney transplantation, Acute pancreatitis, Mycophenolate mofetil

## Abstract

Acute pancreatitis is a rare life-threatening complication in patients after kidney transplantation. Here we described a 56-year-old man who had received a living related kidney transplant for an end-stage renal disease. In his regular follow-up, his serum creatinine was gradually increased and he underwent an allograft biopsy, which revealed an interstitial nephritis/tubular atrophy grade II. Mycophenolate mofetil (MMF) was prescribed to control chronic allograft nephropathy. He presented with complaints of severe abdominal pain, vomiting, loss of appetite and fever requiring hospital admission twelve days later. Acute pancreatitis was diagnosed on the basis of laboratory data and imaging findings during hospital admission. There was no history of alcohol consumption in our patient. Unfortunately he died one week later and autopsy findings demonstrated acute necrotizing pancreatitis. The bladder drainage of this patients was normal. Laboratory findings in this patient did not endorse infections and other possibilities regarding the etiology of acute pancreatitis in this patient. Therefore, we concluded that acute pancreatitis in near the patient was induced by drugs and basis on our evidence, MMF is the most important suspect. This study suggests that acute pancreatitis can be considered as a side effect of MMF.

**Implication for health policy/practice/research/medical education:**

This paper is about an adverse effect of mycophenolate mofetil, acute pancreatitis that should be considered as a rare side effect of this medication.

## Introduction

Acute pancreatitis as a complication after kidney transplant was first reported in 1994 (1). Although this severe complication is rare, has a high mortality rate (2-6). Infection, bladder drainage problems, drugs and alcohol can result in allograft pancreatitis (7,8). Mycophenolate mofetil (MMF) is an immunosuppressive agent that used as prevention for acute rejection, especially within the first 6 months after kidney transplantation (9). The effects of MMF on bone marrow and gastrointestinal toxicity are the most common side effects of this drug (10). MMF induced severe gastrointestinal complications is rare (11). Here we described a case of acute pancreatitis after taking MMF after kidney transplantation.

## Case presentation

A 56-year-old man had received a living related kidney transplant for an end stage renal disease of unknown etiology in 1997. The patient was on triple immunosuppression with steroids, azathioprine, and cyclosporine and had stable graft function. He was followed-up regularly in the renal transplant clinic and the immediate post-transplant period was uneventful. In 2001, the serum creatinine was gradually increased to 3 mg/dl. He underwent an allograft biopsy, which revealed an interstitial nephritis/tubular atrophy grade II. The cyclosporine dosage was reduced (150 mg/day given twice daily) to minimize the chronic nephrotoxicity and MMF (2 g/day in two divided doses) was substituted for azathioprine to control chronic allograft nephropathy. At that time, the patient was afebrile and had a normal physical examination. After conversion, serum creatinine level fell to 2.5 mg/dl. However, he presented with complaints of severe abdominal pain, vomiting, loss of appetite and fever requiring hospital admission twelve days later. MMF was withdrawn but cyclosporine and prednisolone were continued.

On admission, physical examination revealed a moderately dehydrated patient with temperature of 37.8°C, blood pressure of 105/55 mm Hg, heart rate of 110 beats/min, and respiratory rate of 22 breaths/min. Abdominal examination showed a moderately distended abdomen with epigastric tenderness, but no mass or rigidity. Laboratory tests demonstrated a serum amylase level of 1550 U/L (normal range, 30 U/L to 110 U/L), and a serum lipase concentration of 364 U/L (normal range, 5.6 U/L to 51.3 U/L). There was a mild rise in serum creatinine value (2.8 mg/dl). A complete blood count showed a white blood cell count of 11.4×10^9^/L with 65% neutrophils, a hematocrit level of 32.6%. Liver transaminases, total bilirubin, alkaline phosphatase, glucose, lipid profile and electrolytes tests were within the normal ranges. Evaluation for infectious etiology revealed a negative work-up for cytomegalovirus (CMV) antigen. Blood testing for CMV and Epstein–Barr virus (EBV), by PCR was also negative. The electrocardiogram showed normal QRS complexes. Computed tomography of the abdomen demonstrated an enlarged and heterogeneous of his pancreas with poorly delineated borders, consistent with acute pancreatitis. His gallbladder, biliary duct system and spleen were normal. Acute pancreatitis was diagnosed on the basis of laboratory data and imaging findings during hospital admission. He had no history of pancreatitis or other its risk factors. The patient was resuscitated with intravenous fluid and cyclosporine was withdrawn. However, he developed hypotension and respiratory failure requiring transfer to the intensive care unit for ventilatory and inotropic support. Unfortunately, his symptoms worsened, and despite an intensive care, his clinical state deteriorated and he died one week later and autopsy findings demonstrated acute necrotizing pancreatitis. There were no evidence of gallstones or cancer.

## Discussion

Acute pancreatitis is a complication after kidney transplant with incidence of 2% to 7% that has 50% to 100 % mortality rate (2-6). This disorder first described in 1964 (1). Immunosuppressive treatment in patients who undergo kidney transplant, induces pathologic changes that lead to more mortality rate. So patients with acute onset abdominal pain should be considered as acute pancreatitis (12).

There are numbers of possibilities about the etiology of acute pancreatitis. Bile duct stones, drugs, infections, alcohol, hyperlipidemia and hyperparathyroidism are the possibilities of acute pancreatitis after kidney transplant (7,8,13). Hyperparathyroidism in patients with long-term dialysis before kidney transplant should be consider as a theory about acute pancreatitis (13). Viral infection especially CMV is another important suspect in these patients (14). Among treatments that cause acute pancreatitis, immunosuppressive medications are the most possible suspects (12).

MMF is an immunosuppressive agent that used commonly after kidney transplantation. MMF was suggested as a replacement for azathioprine in 1995 (9). The most reported side effects for MMF are gastrointestinal and bone marrow toxicity but severe gastrointestinal complications due to MMF are rare (10,11).

## Conclusion

There was no evidence of bladder drainage problems and using alcohol in our patient. Laboratory findings in this patient did not endorse infections and other possibilities about etiology of acute pancreatitis in our patient. Hence, we concluded that acute pancreatitis in our patient was induced by drugs and basis on our evidence, MMF is the most important suspect. Based on our best knowledge, this is the first report of MMF induced acute pancreatitis in Iran. The above report suggests that acute pancreatitis can be considered as a side effect of MMF. Further studies are necessary to confirm this hypothesis.

## Authors’ contribution

BE; designed the study and prepared data. FD; reviewed the literatures and wrote the manuscript. All authors signed the final manuscript.

## Conflicts of interest

The authors declared no competing interests.

## Ethical considerations

Ethical issues (including plagiarism, data fabrication, double publication) have been completely observed by the authors.

## Funding/Support

None.
